# 657. Genomic Insights into Virulence Factors Affecting a Tissue-invasive *Klebsiella pneumoniae* Infection

**DOI:** 10.1093/ofid/ofab466.854

**Published:** 2021-12-04

**Authors:** Takashi Matono, Masatomo Morita, Nodoka Nakao, Yuji Teshima, Ryosuke Yamate, Takamichi Hijikata, Kosuke Hoashi, Yusuke Ohashi, Yuichi Hasegawa, Tomohide Okinaka, Makoto Ohnishi

**Affiliations:** 1 Aso Iizuka Hospital, Iizuka, Fukuoka, Japan; 2 National Institute of Infectious Diseases, Shinjyuku, Tokyo, Japan

## Abstract

**Background:**

Japan is one of the hypervirulent *Klebsiella pneumoniae* (hvKp) endemic areas, resulting in an alarming issue in actual clinical settings. However, little is known regarding key virulence factors responsible for hvKp infection.

**Methods:**

We analyzed *K. pneumoniae* isolates collected between 2017 and 2019, and defined hvKp as a pyogenic infection. Classical *K. pneumoniae* (cKp) involved a non-invasive infection or uncomplicated bacteremia. Isolates belonging to the *K. pneumoniae* species complex were excluded.

**Results:**

We analyzed 112 isolates, including 19 hvKp, 67 cKp, and 26 colonizers, by whole-genome sequencing. Population genomics revealed that the K1-sequence type (ST) 82 clade was distinct from that of K1-ST23 clone (Figure 1). The virulence-gene profiles also differed between K1-ST82 (aerobactin and *rmpA*) and K1-ST23 (aerobactin, yersiniabactin, salmochelin, colibactin, and *rmpA*/*rmpA2*). The K2 genotype was more diverse than that of K1. A neighboring subclade of K1-ST23 (comprising ST29, ST412, ST36, and ST268) showed multidrug-resistance and hypervirulence potentials. Logistic-regression analysis revealed that diabetes mellitus was associated with *K. pneumoniae* infection (odds ratio [OR]: 4.11; 95% confidence interval [CI]: 1.14–14.8). No significant association was found between hvKp diagnosis and clinical characteristics, such as diabetes mellitus or community acquisition (Table 1). The K1 genotype (OR: 9.02; 95% CI: 2.49–32.7; positive-likelihood ratio [LR]: 4.08), *rmpA* (OR: 8.26; 95% CI: 1.77–38.5; positive LR: 5.83), and aerobactin (OR: 4.59; 95% CI: 1.22–17.2; positive LR: 3.49) were substantial diagnostic predictors of hvKp (Table 2).

Figure 1. Phylogenetic distribution of genetic virulence factors in 112 *K. pneumoniae* isolates

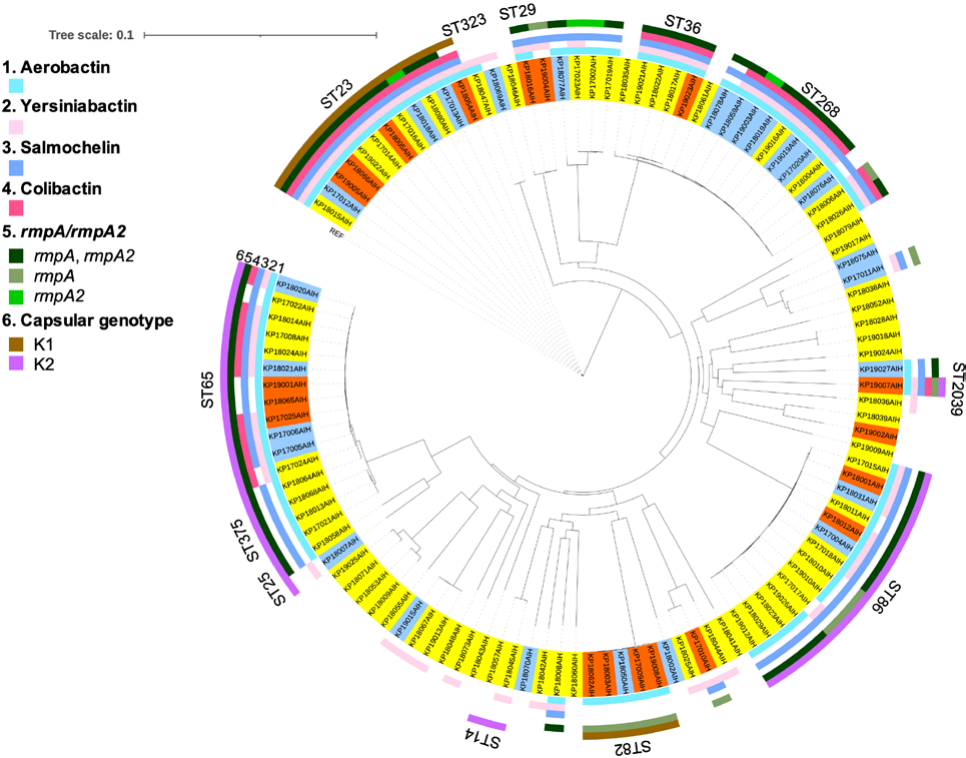

The highlighted strains are clinically pathogenic (orange, hypervirulent *K. pneumoniae*; yellow, classical *K. pneumoniae*; sky blue, colonization). The non-highlighted strain (NTUH-K2044) is a reference *K. pneumoniae* strain.

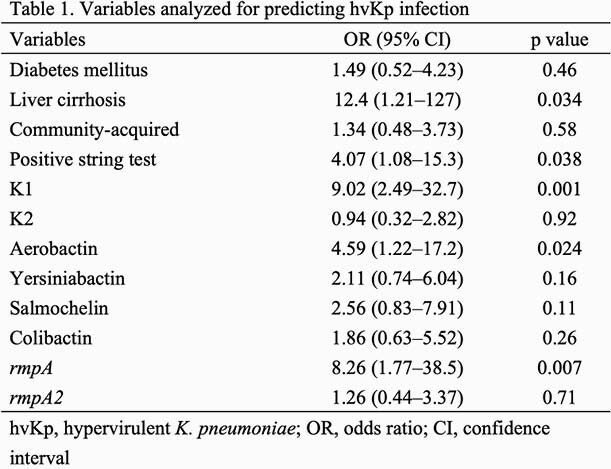

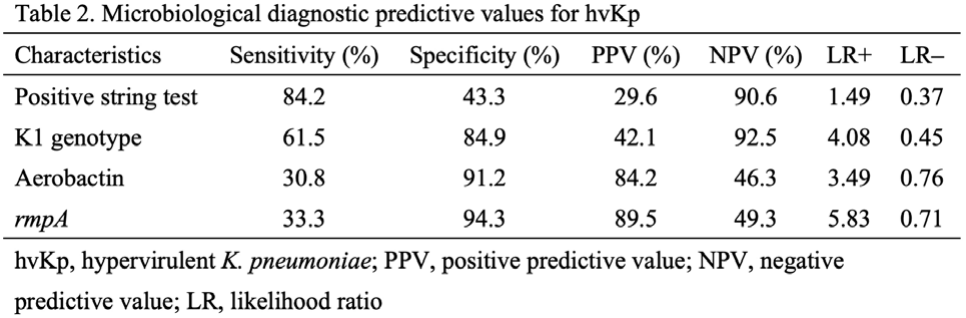

**Conclusion:**

In hvKp-rich settings, diabetes mellitus, community-acquisition, and siderophores other than aerobactin were not remarkable predictors of hvKp infection. However, the K1 genotype, *rmpA*, and aerobactin were found to be substantial predictors, warranting clinical assessment of any possible/further pyogenic (metastatic) infection. We believe that these findings shed light on key hvKp virulence factors.

**Disclosures:**

**All Authors**: No reported disclosures

